# The Late Matron and Worcester General Infirmary

**Published:** 1917-05-26

**Authors:** Mary Herbert

**Affiliations:** 10 Crescent Road, Sidcup, S.E.


### THE EDITOR'S LETTER-BOX.

(*Continued from page* 154.)

### The late Matron and Worcester General Infirmary.

*To the Editor of* The Hospital.

Sir,—A copy of your issue of May 12 has been forwarded to me here, and I read your kind reference to me with much interest and appreciation. May I, however, say that you rather misrepresent my relations towards the authorities, or, rather, theirs towards me? In the earlier part of my time there I had much sympathy and support; and, of course, without it we could have made little or no progress.

They built the nurses' home, and later a fine outpatient department, which made the conditions under which the work was carried on much better in every way.—Believe me, yours faithfully, Mary Herbert.

10 Crescent Road, Sidcup, S.E., May 15, 1917.

